# Visceral fat reference values derived from healthy European men and women aged 20-30 years using GE Healthcare dual-energy x-ray absorptiometry

**DOI:** 10.1371/journal.pone.0180614

**Published:** 2017-07-06

**Authors:** Tomasz Miazgowski, Robert Kucharski, Marta Sołtysiak, Aleksandra Taszarek, Bartosz Miazgowski, Krystyna Widecka

**Affiliations:** Department of Hypertension and Internal Medicine, Pomeranian Medical University, Szczecin, Poland; State University of Rio de Janeiro, BRAZIL

## Abstract

Dual energy X-ray absorptiometry (DXA) is an established technique used in clinical and research settings to evaluate total and regional fat. Additionally, recently developed software allow to quantify visceral adipose tissue (VAT). Currently, there are no reference values available for GE Healthcare DXA systems for VAT. The aim of this study was to develop reference values for VAT in healthy European adults aged 20–30 years using a GE Healthcare Prodigy densitometer along with the dedicated CoreScan application. We also assessed the associations of VAT with traditional cardiometabolic risk factors. In 421 participants (207 men; 214 women), we performed DXA whole-body scans and calculated total body fat (BF) and VAT (in gender-specific percentiles). We also measured blood pressure and fasting glucose, insulin, and blood lipids. Males, in comparison with females, had 2-fold greater VAT both in units of mass (542 ± 451 g; 95% CI: 479.6‒605.1 g vs. 258 ± 226 g; 95% CI: 226.9‒288.6 g) and volume (570 ± 468 cm^3^; 95% CI: 505.1‒635.2 cm^3^ vs. 273 ± 237 cm^3^; 95% CI: 240.6‒305.3 cm^3^). They also had significantly higher the VAT/BF ratio. VAT showed a stronger positive correlation than BF with blood pressure, triglycerides, LDL-cholesterol, glucose, insulin, and homeostasis model assessment-insulin resistance index and a stronger negative correlation with HDL-cholesterol. Among these variables, VAT had the highest area under the curve for triglycerides ≥150 mg/dL (0.727 in males and 0.712 in females). In conclusion, we provide reference values for VAT obtained from healthy adults using the GE Healthcare DXA. These values may be useful in the diagnosis of visceral obesity, for identifying subjects with high obesity-related risks, in epidemiological studies, as a target for therapies, and in physically trained individuals. In both genders, VAT was associated with traditional cardiometabolic risk factors, particularly hypertriglyceridemia.

## Introduction

The excessive accumulation of visceral adipose tissue (VAT) leads to visceral obesity and induces low-grade systemic inflammation, which is mediated by fat-infiltrating immune cells and increased release of proinflammatory cytokines [1–4]. Although the exact mechanisms that initiate VAT accumulation have not been fully elucidated, it is generally believed that excess VAT is closely associated with the development of a cluster of metabolic derangements, hypertension, cardiovascular disease, and malignancies. Visceral obesity can be estimated using several surrogate methods based on anthropometric measures, such as waist circumference, waist-to-hip ratio, waist-to-height ratio, or sagittal abdominal diameter. However, these indices do not allow distinguishing between VAT and subcutaneous abdominal fat and, in general, are fundamentally inaccurate in quantifying VAT [5]. VAT is a relatively small component of total body fat; however, due to known metabolic effects of VAT, there is constantly increasing interest in this fat depot as an attractive target for non-pharmacological [6, 7] and pharmacological interventions [8].

VAT can be accurately measured using magnetic resonance (MRI) and computed tomography (CT) imaging. However, these techniques are costly and may be associated with prolonged scan time or risk of radiation exposure to patients. Therefore, other imaging techniques have been developed to quantify VAT. Of them, dual-energy X-ray absorptiometry (DXA) offers a simple, rapid and accurate estimation of VAT mass and volume [9, 10]. This modality uses the differential attenuation of X-ray beams at two separate energies to calculate the soft tissue composition and can be used to estimate both whole-body and regional distribution of the fat and lean tissues with a relatively low (approximately 1.5 mrem) radiation dose. GE Healthcare and Hologic are the two leading worldwide DXA manufacturers. Recently, both manufacturers enhanced traditional body composition estimated in the whole-body scan by dedicated applications, which automatically calculate VAT by subtracting abdominal subcutaneous fat from total abdominal fat. VAT measured by DXA showed a strong correlation with VAT measured both by CT (R^2^ = 0.957) [9] and MRI (R^2^ = 0.82 for females; R2 = 0.86 for males) [10]. However, there are two important limitations in comparing VAT measures using CT, MRI, and DXA. Firstly, all the modalities quantify VAT in different units–area (cm^2^), volume (cm^3^), or mass (g), making interpretation of results difficult. Secondly, there are no age-, gender-, and race-specific, universally recognized standards for key VAT variables in healthy adults measured by each of these methods, including DXA.

As GE Healthcare DXA is widely used in clinical practice and research investigations, reference standards are needed to define visceral obesity and to evaluate the cardiometabolic risks associated with excess VAT quantified by this instrument. The aim of this study was to develop reference values for VAT mass and volume in healthy young adults using the GE Healthcare Lunar Prodigy instrument along with the dedicated CoreScan application. Additionally, we assessed the associations of DXA-VAT with traditional cardiometabolic risk factors and a surrogate measure of insulin resistance.

## Material and methods

### Study participants

All participants were residents of a large urban area in northwestern Poland. The study population was recruited from March 2014 to June 2016 from 1) participants of the national health-related program evaluating the prevalence of metabolic obesity among the young Polish population (N = 162); this group was randomly selected based on the local electoral roll as described elsewhere [11]; 2) volunteers from university students recruited by local announcements (N = 182); and 3) self-referrals to the Densitometry Unit of the Pomeranian Medical University (N = 77). The inclusion criteria included the following: age between 20 and 30 years, lack of medical conditions that required pharmacological or other treatments, regular menstruation in females, no history of malignancy, abnormal glucose tolerance or rapid weight changes (above 3.0 kg) within the last 12 months, and no apparent abnormalities in the routine physical examination. Due to DXA-specific technical limitations, participants were excluded if their width exceeded the scanner field or their weight exceeded the limits of the scanner bed. Overall, we included 421 participants (207 men; 214 women).

The study complied with all applicable institutional and governmental regulations regarding to the ethical use in human volunteers and with the terms of the Declaration of Helsinki. The Pomeranian Medical University Ethics Committee approved the study protocol, and all the recruited participants gave their written consent.

### Procedures

In all participants, we measured height, weight, and waist and hip circumferences. Blood pressure was measured at least two times in a sitting position using an automated meter, in accordance with current guidelines [12]. Using routine automated methods, we measured fasting plasma glucose, insulin, low- (LDL) and high-density (HDL) lipoproteins, and triglycerides. From insulin and glucose measurements, a homeostasis model assessment-insulin resistance (HOMA-IR) index was calculated. We used the HOMA-IR value of 2.5 as a cutoff for the risk of metabolic syndrome in non-diabetic population [13]. From waist circumference and triglycerides, the lipid accumulation product (LAP) was calculated using the following formulas: LAP = (Waist circumference– 65) x triglycerides [mM/L] in males; and LAP = (Waist circumference– 58) x triglycerides [mM/L] in females. LAP has been shown as a surrogate index of abnormal metabolic profile [14]. Based on the International Diabetes Federation (IDF) race- and gender-specific diagnostic criteria for metabolic syndrome [15], we evaluated the presence of the following risk factors: 1) waist circumference ≥94 cm in men and ≥80 cm in women (for the European population); 2) systolic blood pressure ≥130 mm Hg or diastolic blood pressure ≥85 mm Hg; 3) raised triglyceride level ≥150 mg/dL (1.7 mmol/L); 4) raised fasting plasma glucose ≥100 mg/dL (5.6 mmol/L); and 5) reduced HDL-cholesterol level <50 mg/dL (1.29 mmol/L) in women and <40 mg/dl (1.03 mmol/L) in men.

Body composition parameters, including bone mineral content, lean mass, total body (BF), and android and gynoid fat, were measured by GE-Healthcare Lunar Prodigy Advance (software enCORE; version 14.1) using the automatic whole-body scan mode. All scans were performed and analyzed by a single trained technician per a standard protocol provided by the manufacturer. From BF measures, fat mass index (FMI) as BF (kg) divided by height (m^2^) was calculated (normal ranges: 3‒6 kg/m^2^ in males and 5‒9 kg/m^2^ in females at age 25 [16]). VAT expressed both in grams and cm^3^ was calculated automatically by the CoreScan application. The software algorithm works through detection of the width subcutaneous fat layer within android region of interest on the lateral part of abdomen and the interior-posterior thickness of the abdomen, which can be assessed using X-ray attenuation. From VAT measures, we calculated the following ratios: VAT/BF, VAT/weight, and VAT/Lean.

Instrument quality control was performed on a regular basis using the manufacturer’s block phantom scanned every working day and the Hologic Spine Phantom scanned three times per week. There was no significant drift in calibration for the study period.

### Statistical analysis

Descriptive measures were reported as means ± standard deviation (SD). Data were checked for normality using the Shapiro-Wilk’s test. In the case of normal distribution, means were compared using Student’s t-test; otherwise, the non-parametric Mann-Whitney U-test was used. Chi-square test for independence with Yates’ correction was used to determine if qualitative variables were related. The relationship between pairs of quantitative variables with normal distribution was presented using Pearson’s linear correlation coefficient, whereas Spearman’s rank correlation coefficient was calculated for pairs with non-normal distribution. VAT was calculated in the units of mass (g) and volume (cm^3^) and presented as sex-specific percentiles. Quantile regression coefficients were computed to compare each VAT percentile between males and females. A receiver operating characteristic (ROC) curve analysis was used to assess the accuracy for each component of metabolic syndrome defined by the IDF criteria [15], LDL-cholesterol, and HOMA-IR. The accuracy was measured using the area under the curve (AUC) with a 95% confidence interval (CI). To determine the appropriate gender-specific cut-off point for VAT, the score with the highest combination of sensitivity and specificity (Youden’s index, sensitivity + specificity ‒ 1) was considered the optimal cut-off score. Statistical analyses were performed using SPSS version 23.0 and R Statistics version 3.3.2 (available from: www.cran.r-project.org).

## Results

Baseline characteristics of studied participants are shown in Table 1.

**Table 1:** Descriptive characteristics of participants by gender.

	All (N = 421)	Males (N = 207)	Females (N = 214)	P value males vs. females
Age (years)	26.52 ± 3.18	25.04 ± 3.06	27.95 ± 2.59	<0.001
Height (cm)	173.43 ± 10.42	181.80 ± 6.98	165.34 ± 5.75	<0.001
Weight (kg)	70.94 ± 13.88	81.03 ± 11.17	61.23 ± 8.11	<0.001
Body Mass Index (kg/m^2^) ≤18.4 18.5–24.9 25.0–29.9 ≥30.0	23.37 ± 2.74 5 (1.2%) 328 (77.9%) 79 (18.7%) 9 (2.1%)	24.46 ± 2.65 0 139 (67.1%) 64 (30.9%) 4 (1.93%)	22.33 ± 2.40 5 (2.3%) 189 (88.3%) 15 (7.0%) 5 (2.3%)	<0.001 0.026 <0.001 <0.001 0.082
Waist circumference (cm)	81.72 ± 10.09	87.35 ± 9.07	76.28 ± 7.79	<0.001
Hip circumference (cm)	95.43 ± 6.78	95.46 ± 7.08	95.40 ± 6.50	0.936
Waist-to-hip ratio	0.86 ± 0.09	0.92 ± 0.07	0.80 ± 0.07	<0.001
Total body fat (g)	19491.6 ± 6260.8	19201.77 ± 7066.9	19770.39 ± 5375.6	0.361
Total body fat (%)	28.23 ± 7.25	24.16 ± 6.55	32.15 ± 5.53	<0.001
Lean mass (g)	48753.9 ± 11455	58671.06 ± 7001.8	39216.50 ± 4944.8	<0.001
Android fat (g)	1439.94 ± 768.13	1558.46 ± 886.49	1325.95 ± 614.75	0.002
Gynoid fat (g)	3826.29 ± 1500.4	3223.77 ± 1151.32	4408.53 ± 1569.54	<0.001
VAT (cm^3^)	418.72 ± 397.13	570.24 ± 467.75	273.00 ± 237.09	<0.001
VAT (g)	397.27 ± 381.82	542.31 ± 451.09	257.78 ± 226.11	<0.001
VAT/BF ratio (%)	1.85 ± 1.36	2.55 ± 1.48	1.18 ± 0.78	<0.001
VAT/Weight ratio (%)	0.52 ± 0.42	0.64 ± 0.47	0.40 ± 0.31	<0.001
VAT/Lean ratio (%)	0.78 ± 0.67	0.92 ± 0.75	0.65 ± 0.55	<0.001
Fat Mass Index (kg/m^2^)	6.51 ± 2.11	5.80 ± 2.08	7.21 ± 1.89	<0.001
Glucose (mg/dL)	89.42 ± 7.77	89.98 ± 8.03	89.07 ± 7.60	0.297
Insulin (IU/mL)	7.27 ± 3.72	8.19 ± 3.67	6.72 ± 3.65	0.001
HOMA-IR	1.61 ± 0.86	1.82 ± 0.82	1.48 ± 0.86	0.001
Triglycerides (mg/dL)	84.00 ± 41.65	102.54 ± 50.82	72.51 ± 29.54	<0.001
Lipid Accumulation Product	21.08 ± 18.24	30.10 ± 23.22	15.46 ± 11.11	<0.001
HDL-cholesterol (mg/dL)	60.43 ± 15.53	53.54 ± 12.75	64.69 ± 15.60	<0.001
LDL-cholesterol (mg/dL)	105.89 ± 31.16	109.12 ± 30.81	103.90 ± 31.28	0.137
Systolic blood pressure (mm Hg)	124.14 ± 16.95	131.38 ± 16.38	117.01 ± 14.29	<0.001
Diastolic blood pressure (mm Hg)	77.38 ± 9.58	78.57 ± 9.35	76.21 ± 9.69	0.015

The mean age of the sample was 26.5 ± 3.2 years (range: 20.1‒30.0 years) and BMI ranged from 17.1 to 40.2 kg/m^2^. Based on the BMI classification, 78% of participants had normal body weight. The frequency of underweight was higher in females, while overweight was more frequent among males. The mean values of FMI in males and females were within normal reference ranges at age 25 [16].

In comparison with females, males had lower BF% and gynoid fat but higher lean mass, android fat, and VAT mass and volume. VAT was a relatively small component of the body and accounted for only 2.6% and 1.2% of BF and 0.6% and 0.4% of weight in men and women, respectively. The VAT/Lean ratio was also greater in men. In both genders, mean values of IDF-metabolic syndrome components as well as fasting insulin and HOMA-IR were within normal ranges. However, despite similar fasting glucose levels in both genders, males had higher fasting insulin concentration and HOMA-IR. They also had significantly higher blood pressure, triglyceride level, and LAP calculated from triglycerides and waist circumference.

The mean values and percentiles (from the 10^th^ to the 90^th^) of VAT and the VAT/BF ratios for males and females are displayed in Table 2. Males, in comparison with females, had two times greater VAT in both units of mass (542 ± 451 g vs. 258 ± 226 g) and volume (570 ± 468 cm^3^ vs. 273 ± 237 cm^3^). They also had significantly higher VAT/BF ratios (p<0.001).

**Table 2:** Sex-specific percentiles of VAT and VAT/BF ratio.

	Mean	SD	95% CI	10^th^	20^th^	30^th^	40^th^	50^th^	60^th^	70^th^	80^th^	90^th^
Males												
VAT (g)	542.31	451.09	479.6; 605.1	103.4	187.4	251.8	307.8	391.0	484.8	676.8	891.4	1259.4
VAT (cm^3^)	570.24	467.75	505.1; 635.2	109.4	198.4	266.8	325.8	414.0	514.0	717.2	945.0	1335.6
VAT/BF Ratio	2.55	1.48	2.34; 2.75	0.71	1.20	1.63	2.11	2.34	2.70	3.17	3.83	4.55
Females												
VAT (g)	257.78	226.11	226.9; 288.6	20.0	73.0	125.0	162.0	204.0	258.0	324.0	405.0	515.0
VAT (cm^3^)	272.99	237.09	240.6; 305.3	18.0	76.0	133.0	172.0	216.0	273.0	343.0	429.0	545.0
VAT/BF Ratio	1.18	0.78	1.08; 1.29	0.12	0.51	0.74	0.91	1.07	1.32	1.55	1.82	2.14

P < 0.001 for comparisons between males and females in the mean values and each percentile

As summarized in Table 3, in contrast to BF, VAT and the VAT/BF ratio did not correlate with age. Moreover, VAT showed a stronger positive correlation than BF with insulin, glucose, HOMA-IR, systolic and diastolic blood pressure, LAP, and blood lipids (especially triglycerides) (positively) and a stronger negative correlation with HDL-cholesterol. Like VAT, the VAT/BF ratio showed moderate to strong correlations with most cardiometabolic risk factors.

**Table 3:** Correlation coefficients between VAT, body fat, anthropometric indices, blood pressure, blood lipids and HOMA-IR (males and females combined).

Variables	VAT (g)	VAT (cm^3^)	BF (g)	BF (%)	VAT/BF ratio
Age (years)	0.096	0.097	0.186 ^c^	0.378 ^c^	0.028
Weight (g)	0.655 ^c^	0.659 ^c^	0.523 ^c^	- 0.072	0.605 ^c^
Waist circumference (cm)	0.758 ^c^	0.761 ^c^	0.635 ^c^	0.162 ^c^	0.666 ^c^
Hip circumference (cm)	0.412 ^c^	0.413 ^c^	0.630 ^c^	0.401 ^c^	0.243 ^c^
Waist-to-hip ratio	0.604 ^c^	0.607 ^c^	0.315 ^c^	- 0.089	0.617 ^c^
Lean mass(g)	0.390 ^c^	0.391 ^c^	0.091	- 0.501 ^c^	0.461 ^c^
VAT/Lean ratio (%)	0.960 ^c^	0.960 ^c^	0.728 ^c^	0.494 ^c^	0.873 ^c^
Android fat (g)	0.827 ^c^	0.831 ^c^	0.908 ^c^	0.641 ^c^	0.624 ^c^
Gynoid fat (g)	0.238 ^c^	0.240 ^c^	0.676 ^c^	0.749 ^c^	0.011
Body Mass Index (kg/m^2^)	0.716 ^c^	0.718 ^c^	0.726 ^c^	0.294 ^c^	0.580 ^c^
Fat Mass Index (kg/m^2^)	0.536 ^c^	0.539 ^c^	0.926 ^c^	0.930 ^c^	0.265 ^c^
Systolic blood pressure (mm Hg)	0.289 ^c^	0.289 ^c^	0.078	0.170 ^b^	0.327 ^c^
Diastolic blood pressure (mm Hg)	0.247 ^c^	0.247 ^c^	0.191 ^c^	0.095	0.211 ^c^
Triglycerides (mm Hg)	0.525 ^c^	0.526 ^c^	0.330 ^c^	0.081	0.506 ^c^
Lipid Accumulation Product	0.767 ^c^	0.769 ^c^	0.613 ^c^	0.244 ^c^	0.676 ^c^
HDL-cholesterol (mg/dL)	- 0.361 ^c^	- 0.364 ^c^	- 0.250 ^c^	- 0.023	- 0.375 ^c^
LDL-cholesterol (mg/dL)	0.295 ^c^	0.295 ^c^	0.251 ^c^	0.185 ^c^	0.239 ^c^
Glucose (mg/dL)	0.126 ^a^	0.125 ^a^	0.022	- 0.002	0.124 ^a^
Insulin (IU/mL)	0.410 ^c^	0.418 ^c^	0.378 ^c^	0.190 ^c^	0.337 ^c^
HOMA-IR	0.396 ^c^	0.403 ^c^	0.360 ^c^	0.175 ^b^	0.331 ^c^

^a^ P<0.05

^b^ P<0.01

^c^ P<0.001

We next attempted to calculate gender-specific VAT cutoffs for the analyzed cardiometabolic risk factors. In the AUC analysis, VAT in both sexes was a weak to moderate predictor of most IDF-metabolic syndrome components, LDL-cholesterol, and insulin resistance evaluated by HOMA-IR (Table 4).

**Table 4:** AUC values and cut-off points for VAT volume by gender.

	AUC	95% CI	P value	Cut off	Sensitivity	Specificity	Younden’s Index
Systolic blood pressure ≥130 mm Hg							
Males	0.628	0.501; 0.722	0.001	596	0.460	0.721	0.181
Females	0.611	0.496; 0.804	0.001	399	0.521	0.782	0.303
Diastolic blood pressure ≥85 mm Hg							
Males	0.621	0.525; 0.812	0.001	331	0.464	0.722	0.186
Females	0.619	0.491; 0.768	0.001	161	0.486	0.734	0.112
Waist circumference							
Males ≥94 cm	0.914	0.856; 0.963	0.001	762	0.809	0.890	0.698
Females ≥80 cm	0.839	0.776; 0.901	0.001	256	0.821	0.752	0.538
LDL-cholesterol ≥100 mg/dl							
Males	0.628	0.513; 0.901	0.001	672	0.424	0.865	0.289
Females	0.636	0.544; 0.827	0.001	326	0.468	0.820	0.288
HDL-cholesterol							
Males <40 mg/dl	0.672	0.552; 0.831	0.004	759	0.694	0.688	0.372
Females <50 mg/dl	0.659	0.543; 0.755	0.002	345	0.581	0.756	0.336
Glucose ≥100 mg/dl							
Males	0.633	0.541; 0.725	0.004	812	0.469	0.734	0.265
Females	0.621	0.523; 0.628	0.004	408	0.498	0.725	0.227
Triglycerides ≥150 mg/dl							
Males	0.787	0.681; 0.873	0.001	762	0.773	0.724	0.497
Females	0.737	0.595; 0.879	0.036	229	1.0	0.555	0.555
HOMA-IR ≥2.5							
Males	0.727	0.638; 0.816	0.001	1082	0.405	0.771	0.366
Females	0.712	0.611; 0.806	0.001	499	0.512	0.840	0.328

**Fig 1:**
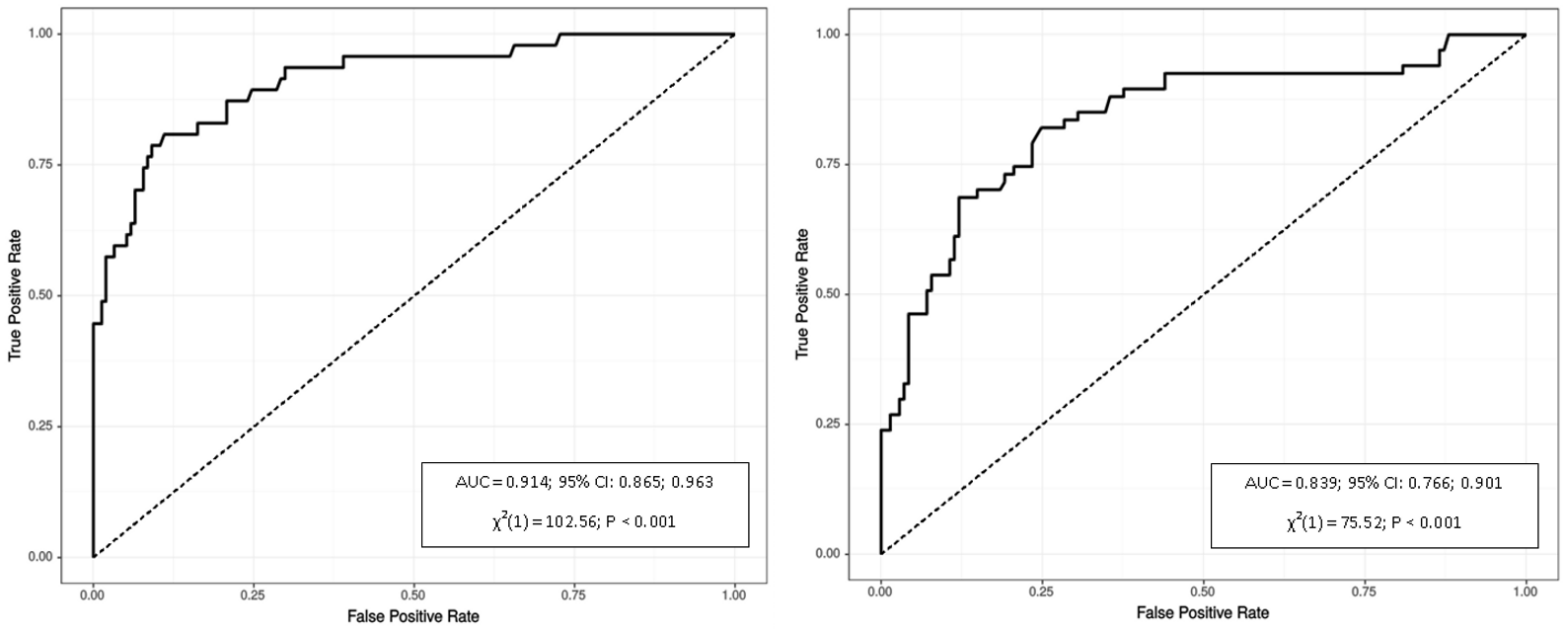
Receiver operating characteristics of VAT for identifying waist circumference ≥94 cm in males (left) and ≥80 cm in females (right). P refers to logistic regression model.

**Fig 2:**
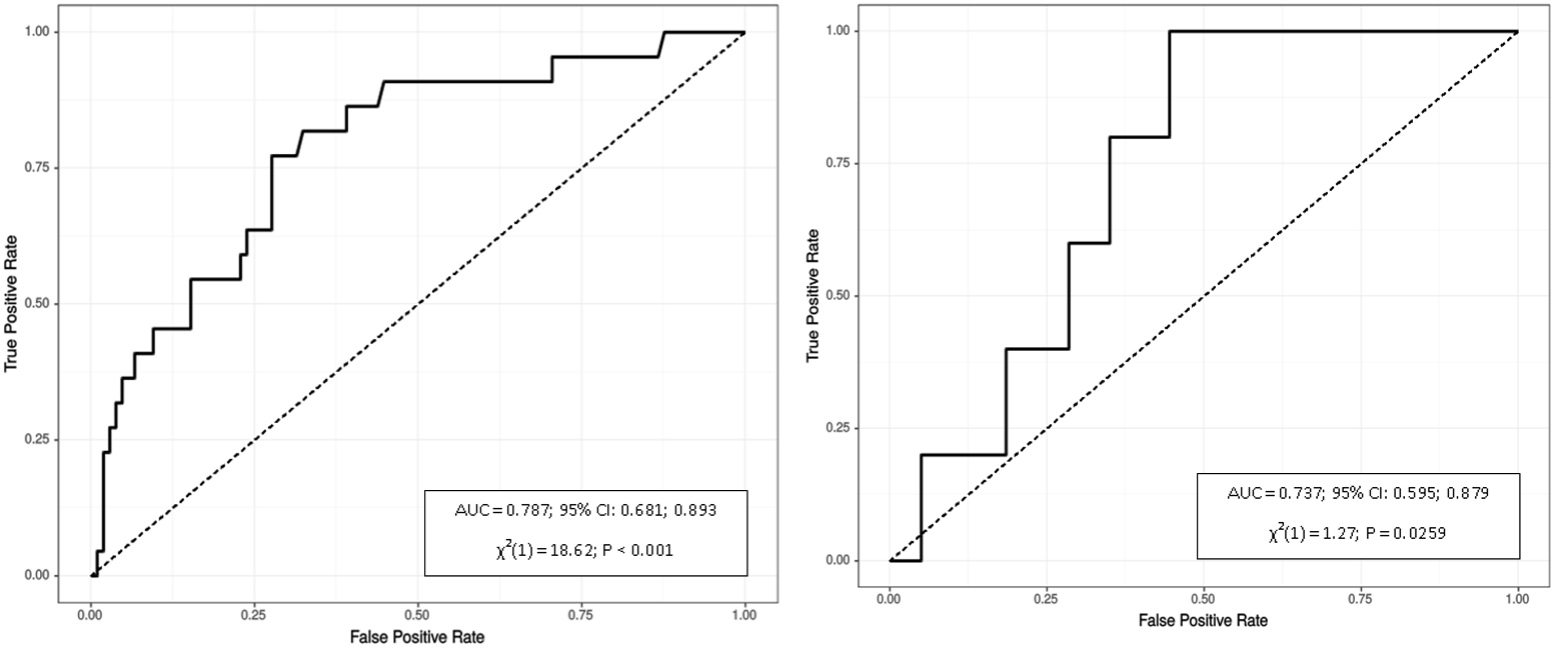
Receiver operating characteristics of VAT for identifying triglyceride level ≥150 mg/dL in males (left) and females (right). P refers to logistic regression model.

However, values of the Youden’s Index, which was used as a measure of quality for the definition of the optimal cutoffs, were relatively low for these factors. As expected, VAT had the highest area under the curve for gender-specific waist circumference (AUX = 0.914 for males and 0.839 for females), which corresponded to the values above the 70^th^ and 50^th^ percentiles of VAT volume for men and women, respectively. VAT was also significantly associated with triglyceride level. The VAT cutoffs predicting triglycerides ≥150 mg/dL were similar to those predicting an elevated waist circumference ≥94 cm in males and ≥80 cm in females (761 cm^3^ and 239 cm^3^, respectively). ROC curve analyses of VAT for prediction of abdominal obesity defined by waist circumference and hypertriglyceridemia are displayed in Fig 1 and Fig 2, respectively.

## Discussion

In this cross-sectional study using the GE Healthcare Lunar Prodigy densitometer, we developed reference values for VAT derived from a homogenous group of healthy European adults aged 20–30 years. These reference values may be useful for identifying subjects with excess visceral fat and high obesity-related risks, in epidemiological studies, as a target for therapies, and in physically trained individuals. However, whether the definition of visceral obesity based on the cutoffs calculated in this study is useful and appropriate requires further investigation. Future research should look at visceral obesity-related morbidity and outcomes using the same modality. This is because body composition is not only influenced by sex, age, geographic location, and ethnicity [16–19] but also the method of assessment. The VAT indices measured by CT, MRI, and DXA, although strongly correlated, may differ both in absolute values and type of units as they may be expressed in units of mass, area, or volume. Even if the same method is used but the measures are performed on instruments from different manufacturers, the results may vary significantly. Regarding DXA, inter-device differences in body composition between two dominant manufacturers (GE Healthcare and Hologic) have been demonstrated [20, 21], suggesting possible similar inter-machine differences in assessing VAT. Therefore, DXA-VAT reference values should be developed as specific for each manufacturer until they are cross-validated.

To our best knowledge, this study is the first report providing reference standards for VAT measured by Lunar Prodigy and CoreScan software in healthy European population aged 20–30 years. We found that males had 2-fold greater VAT, expressed both in units of mass (542 ± 451 g vs. 258 ± 226 g) and volume (570 ± 468 cm^3^ vs. 273 ± 237 cm^3^), than healthy females. Similarly, the VAT-to-fat, VAT-to-weight, and VAT-to-Lean ratios were significantly higher in males. In our previous research [22], we determined VAT by the same machine in the population of lean women (BMI <25.0 kg/m^2^) aged 20–40 years and found slightly lower VAT mass (236 ± 183 g) and volume (250 ± 195 cm^3^) in comparison with women in the current study. As in both studies VAT was strongly correlated with BF%, these differences may reflect a positive relationship of VAT with total adiposity. Such a relationship was also suggested by other reports [4, 23].

We found that VAT correlated more strongly than BF with all evaluated cardiometabolic risk factors, including blood pressure, lipids, insulin and glucose, and HOMA-IR. Previous reports univocally demonstrated the association of excess VAT with the risk of cardiovascular and metabolic disorders, which are primarily driven by insulin resistance [1, 4, 13, 17, 18, 24]. Our findings suggest that, even in young and apparently healthy individuals, VAT might be an early marker of hypertension, atherogenic lipid profiles, and insulin resistance. It has been suggested that the accumulation of triglycerides and free fatty acids in the adjacent abdominal organs (i.e., in the liver and pancreas) as a result of increased lipolysis induced by VAT plays a crucial role in the development of insulin resistance [25, 26]. Our results seem to confirm this scenario, because in males and females without known metabolic diseases, VAT was strongly correlated with triglyceride levels, and VAT values above the 70^th^ percentile in males and the 50^th^ percentile in females were robust predictors of hypertriglyceridemia. Similar conclusion may be drawn from earlier reports [4, 17].

Our study has some limitations. Firstly, we assessed healthy Polish population aged 20–30 years and therefore, the presented reference standards for VAT may not apply to other populations. Second, there was over-representation in our cohort of normal weight subjects (78%) and relatively a low number of overweight and obese. This was partially caused by technical limitations in performing the whole-body scan in subjects whose body size or weight exceed the DXA limits. Finally, our reference values were developed using GE the Healthcare Lunar Prodigy densitometer and hence, they may not apply to VAT obtained by other methods, including DXA from other manufacturers. However, they may be used when comparing VAT obtained by another GE Healthcare device, iDXA. Both Prodigy and iDXA use the same CoreScan application to quantify VAT. In addition, studies comparing DXA-VAT measured by Prodigy and iDXA showed a similar precision and good agreement between both devices [27]. This study had some strengths, including the strictly selected homogenous population studied across a range of BMIs and FMIs. Additionally, all whole-body DXA scans were analyzed by a single technician and all of them required no manual correction for the accuracy of android and gynoid regions of interest, which minimized observer error.

In conclusion, the results from this study provide reference values for VAT obtained from a homogenous group of healthy adults using the GE Healthcare Lunar Prodigy instrument. In both genders, VAT was associated with traditional cardiometabolic risk factors, particularly hypertriglyceridemia.

## Supporting information

S1 Database VAT Patient database. (XLSX)
